# Vitamin D concentrations in familial combined hyperlipidemia: effects of lipid lowering treatment

**DOI:** 10.1186/1758-5996-6-7

**Published:** 2014-01-22

**Authors:** Inka Miñambres, Jose Luis Sánchez-Quesada, Joan Sánchez-Hernández, Jose Rodríguez, Alberto de Leiva, Antonio Pérez

**Affiliations:** 1 Endocrinology and Nutrition Department, Hospital de la Santa Creu i Sant Pau, C/Sant Antoni Maria Claret 167, 08025 Barcelona, Spain; 2 Medicine Department, Universitat Autònoma de Barcelona (UAB), C/Sant Antoni Maria Claret 167, 08025 Barcelona, Spain; 3 Biomedical Research Institute IIB Sant Pau. Barcelona, C/Sant Antoni Maria Claret 167, 08025 Barcelona, Spain; 4 Diabetes and Metabolic Diseases CIBER (CIBERDEM), C/Sant Antoni Maria Claret 167, 08025 Barcelona, Spain; 5 Clinical Biochemistry Department, Hospital de la Santa Creu i Sant Pau, C/Sant Antoni Maria Claret 167, 08025 Barcelona, Spain; 6 Bioengineering, Biomaterials and Nanomedicine CIBER (CIBERBBN), C/Sant Antoni Maria Claret 167, 08025 Barcelona, Spain

**Keywords:** Vitamin D, Dyslipidemia, Familial combined hyperlipidemia, Metabolic syndrome, Insulin resistance

## Abstract

**Background:**

Vitamin D deficiency has been linked to several cardiovascular risk factors but information regarding vitamin D concentrations in familial combined hyperlipidemia (FCHL) is lacking. Our objective was to examine vitamin D concentrations in patients with FCHL and to study the effects of lipid-lowering therapy.

**Methods:**

We conducted a cross sectional study on 59 patients with FCHL and 48 healthy controls. We analyzed 25-hydroxyvitamin D (25(OH)D) concentrations and their association with lipid parameters, anthropometric measures, C-reactive protein and homeostasis model assessment (HOMA) index. Twenty-three patients with FCHL were also included in a longitudinal study conducted to analyze 25-hydroxyvitamin D concentrations before and after treatment for dyslipidemia.

**Results:**

After adjustment for body mass index and seasonality, patients with FCHL had lower vitamin D concentrations than controls. Adjusted means (standard error of the mean (S.E.M)) for 25(OH)D according to the presence or absence of FCHL were 62.8 (3.6) nmol/L for patients with FCHL and 74.8 (4.1) nmol/L for controls (p = 0.021). In FCHL, hypovitaminosis D was associated with features of atherogenic dyslipidemia. After lipid-lowering therapy, vitamin D concentrations increased (51.0 ± 31.3 to 58.9 ± 24.6 nmol/L (P = 0.022)). However, changes in 25(OH)D concentrations did not correlate with changes in other parameters.

**Conclusions:**

Our findings suggest that FCHL is associated with decreased vitamin D concentrations and that treatment for dyslipidemia improves vitamin D status through an unknown mechanism. Further studies are needed to replicate these data in larger populations and to elucidate the mechanisms involved in this association.

## Introduction

There is increasing evidence that vitamin D may have an effect on cardiovascular outcomes. Many reports have pointed towards an association of vitamin D deficiency with the occurrence of fatal and non-fatal cardiovascular events [1-3] and with several cardiovascular risk factors such as hypertension, diabetes or dyslipidemia [4-9]. Special attention has been paid to the association of vitamin D deficiency with insulin resistant conditions, such as obesity or the metabolic syndrome [10-15].

Familial combined hyperlipidemia (FCHL) is a complex genetic disorder characterized by variable phenotypic expression and associated with obesity and insulin resistance [16,17]. It is characterized by hypertriglyceridemia, hypercholesterolemia, reduced high-density lipoprotein cholesterol (HDL-c), increased apolipoprotein B (apoB) and predominance of small, dense low-density lipoprotein (LDL) particles, features that are also present in conditions of insulin resistance [18,19]. FCHL is the most common familial form of hyperlipidemia and carries a high risk of cardiovascular disease.

We hypothesized that FCHL could be associated with low concentrations of vitamin D as occurs with other conditions linked to insulin resistance. To test our hypothesis, we compared vitamin D concentrations in patients with FCHL with those from healthy controls. Moreover, we studied the association of vitamin D concentrations with anthropometric characteristics, lipoprotein parameters, C-reactive protein (CRP) and insulin resistance and analysed the effects of lipid-lowering treatment.

## Methods

In a previous study we included 113 patients with FCHL and 54 normoglycemic and normolipidemic healthy subjects in order to investigate qualitative lipoprotein modifications in FCHL. Patients were recruited between 2007 and 2008 at a tertiary care centre in Spain. Criteria for FCHL diagnosis were having at least one first-degree relative with hyperlipidemia and having off-treatment serum LDL cholesterol (LDL-c) and/or triglyceride levels above the gender- and age-specific 90^th^ percentiles of the Spanish reference population [20]. If the patient had type 2 diabetes, the above criteria and the presence of LDL-c levels > 4.14 nmol/L were mandatory. The presence of secondary causes of dyslipidemia was an exclusion criteria. Controls were recruited within hospital employees and their relatives and were included in the study if they had not been previously diagnosed with disturbances of lipid or glucose metabolism and had glucose concentrations < 5,6 mmo/L, LDL-c concentrations < 4,14 mmol/L and triglyceride concentrations < 2,25 mmol/L*.* For the present study, we selected 59 patients with FCHL and 48 healthy controls for whom frozen serum samples were available in order to determine 25-hydroxyvitamin D (25(OH)D) concentrations. All patients and controls were included in a transversal study. Twenty-three of the 59 patients with FCHL were also included in a longitudinal study and vitamin D concentrations were studied before and after treatment for their dyslipidemia for a minimum of 2 months. All subjects were Caucasian and none were taking vitamin D supplements. The investigation conforms to the principles outlined in the Declaration of Helsinki and was approved by the hospital ethics committee. All patients gave written informed consent prior to participation.

Blood specimens were obtained after overnight fasting. The basic lipid profile was obtained using fresh blood samples. All other analyses were performed using ethylenediaminetetraacetic acid-plasma samples frozen at -80°C. To minimize inter-assay variability, samples from the same subject were analyzed in the same batch.

The lipid profile included total cholesterol, triglycerides, very low-density lipoprotein cholesterol (VLDL-c), LDL-c, HDL-c, apoB, and non-esterified fatty acids (NEFA). Total cholesterol and triglycerides were measured by commercially available enzymatic methods, and HDLc was measured by a direct method. LDLc was calculated using Friedewald’s formula when triglycerides did not exceed 300 mg/dl (3.45 mmol/liter) [21]. Otherwise, ultracentrifugation was performed, and LDLc was estimated in the infranatant after separating the fraction with a density less than 1.006. kg/liter, according NCEP-ATPIII recommendations [22]. VLDLc was calculated by substraction. All reagents were from Roche Diagnostics GmbH, Basel, Switzerland, except for NEFA (Wako Chemicals, Neuss, Germany) and all tests were performed in a Hitachi 917 auto analyzer. LDL size was determined by nondenaturing polyacrylamide gradient (2% to 16%) gel electrophoresis, as previously described [23].

CRP was measured by a highly sensitive commercial method (hsCRP, Roche Diagnostics) in a Hitachi 917 auto analyzer.

In subjects with FCHL, serum insulin concentrations were measured by an automated, solid-phase, two-site chemiluminescent immunometric assay (Immulite 2000, Diagnostic Products Corp., Los Angeles, California, USA), with 8% cross-reactivity with proinsulin and a total analytical imprecision less than 7.5% for values between 55 and 2100 pmol/L (7.7 and 291 mIU/mL). The homeostasis model assessment (HOMA) index was calculated as (glucose (mmol/L) · insulinemia (μUI/mL))/22.5 and insulin resistance was defined as HOMA concentrations > 3.29, based on the 90^th^ percentile of the Spanish population [24,25].

Serum 25(OH)D concentrations were determined using a commercial enzyme immunoassay (Immunodiagnostic Systems Ltd (IDS), Boldon, United Kingdom). Both intra- and inter-assay coefficients of variation (CVs) for serum 25(OH)D were < 6.8% and < 8.8%, respectively, for mean concentrations between 38 and 164 nmol/L. Analytical sensitivity was 5 nmol/L. Previous reports show that 25-hydroxyvitamin D is extremely stable in plasma and that serum degradation of vitamin D has not been proven after 10 years stored in a frozen state [26]. 25(OH)D concentrations were defined as sufficient (>80 nmol/L) or hypovitaminosis D (≤80 nmol/L) based on previous studies regarding the effects of vitamin D on parathyroid hormone, calcium absorption, and bone mineral density [27].

Data were analyzed using the SPSS 18.0 statistical package (SPSS Inc). Normality of data distribution was evaluated using Kolmogorov-Smirnov in the transversal analysis and Shapiro-Wilk test in the longitudinal analysis. The relationship between qualitative variables was assessed using Chi-square test and quantitative variables were analyzed by means of Pearson’s or Spearman’s correlation coefficient. Differences between groups were evaluated by T-test, Mann–Whitney test and analysis of covariance (ANCOVA). For the longitudinal study Mann–Whitney test, paired T-test, Wilcoxon test and Pearson’s or Spearman’s correlation analysis were used. The level of statistical significance was set at p < 0.05.

## Results

### Cross-sectional study

Table 1 shows the clinical characteristics of patients with FCHL and control subjects. Among subjects with FCHL, 5 patients (8.5%) had type 2 diabetes and 21 patients (35.6%) had hypertension. Seventeen patients (28.8%) were taking statins and 6 (10.2%) were taking fenofibrate.

**Table 1:** Clinical and biochemical characteristics of patients with FCHL and control subjects

	**Cases (n = 59)**	**Controls (n = 48)**	**P**
Age (years)	50.2 ± 11.6	46.5 ± 17.2	0.210
Sex (% men)	57.6	62.5	0.609
BMI (Kg/m^2^)	27.9 ± 4.1	25.4 ± 4.1	0.005
Waist (cm)			0.026
- Men	97.7 ± 9	91.79 ± 12.1	
- Women	92.7 ± 12.4	88.8 ± 11.8	
Glucose (mmol/L)	5.3 ± 0.6	4.9 ± 0.73	0.020
HbA1c (%)	5.6 ± 0.5	5.4 ± 0.6	0.135
Total cholesterol (mmol/L)	6.7 ± 1.6	4.8 ± 0.8	0.000
Triglycerides (mmol/L)	2.3 (1.1-15.8)	0.7 (0.3-2.1)	0.000
LDL-c (mmol/L)	4.1 ± 1.3	2.8 ± 0.7	0.000
HDL-c (mmol/L)	1.2 ± 0.4	1.6 ± 0.4	0.000
VLDL-c (mmol/L)	1.0 (0.5-7.6)	0.4 (0.1-1.2)	0.000
NEFA (mmol/L)	0.7 (0.2-4.3)	0.4 (0.1-1.1)	0.000
ApoB (g/L)	1.3 ± 0.3	0.8 ± 0.2	0.000
CRP (mg/L)	1.6 (0.2-21.5)	1.0 (0.2-15.0)	0.133
25(OH)D (nmol/L)	58.9 ± 24.5	69.1 ± 30.0	0.057

P values according to T-test and Mann–Whitney test.

FCHL: Familial combined hyperlipidemia, BMI: body mass index, HbA1c: glicated haemoglobin, LDL-c: low-density lipoprotein cholesterol, HDL-c: high-density lipoprotein cholesterol, VLDL-c: very low-density lipoprotein cholesterol, NEFA: non-esterified fatty acids, ApoB: apolipoprotein B, CRP: C-reactive protein, 25(OH)D: 25-hydroxyvitamin D.

As expected, all lipid parameters were higher in patients with FCHL, except for HDL-c, which was lower. A trend towards lower 25(OH)D concentrations in patients with FCHL was observed. Results from the ANCOVA performed to detect differences in 25(OH)D concentrations between patients and controls after adjusting for seasonality and body mass index (BMI), showed that seasonality (p = 0.007) and the presence or absence of FCHL (p = 0.021) were significant predictors of 25(OH)D concentrations, whereas BMI did not influence them. Adjusted means (standard error of the mean (S.E.M)) for 25(OH)D, according to the presence/absence of FCHL and adjusted for seasonality and BMI, were 62.8 (3.6) nmol/L for patients with FCHL and 74.8 (4.1) nmol/L for controls. Correlation analysis did not show any association between 25(OH)D concentrations and BMI, waist, age, lipid parameters or CRP in controls. However, in subjects with FCHL, calcidiol concentrations correlated with LDL-c (r 0.257; p = 0.049) and triglyceride concentrations (r -0.265; p = 0.042).

Forty- six patients with FCHL (78.8%) presented hypovitaminosis D. Patients with hypovitaminosis D had higher triglyceride and NEFA concentrations and lower LDL-c and HDL-c concentrations than patients with normal 25(OH)D concentrations (Table 2). Differences in triglyceride and NEFA concentrations remained significant after adjustment for seasonality and BMI. Furthermore, patients with triglyceride concentrations > 2.25 nmol/L (200 mg/dL) had lower 25(OH)D concentrations than patients with triglyceride < 2.25 nmol/L (50.63 ± 21.3 vs. 67.49 ± 25 nmol/L; p = 0.007). No differences in 25(OH)D concentrations were found between patients with and without lipid-lowering therapy or between patients with or without insulin resistance.

**Table 2:** Clinical and biochemical characteristics of patients with FCHL according to vitamin D status

	**Deficient (n = 46)**	**Sufficient (n = 13)**	**P**
Age (years)	51.7 ± 10.3	44.9 ± 14.5	0.061
BMI (Kg/m^2^)	28.2 ± 4.0	26.8 ± 4.0	0.289
Waist (cm)	96.2 ± 10.1	93.3 ± 12.8	0.412
HbA1c (%)	5.6 ± 0.5	5.6 ± 0.4	0.838
HOMA	2.7 ± 2.4	1.8 ± 0.9	0.838
Total cholesterol (mmol/L)	6.6 ± 1.6	7.1 ± 1.3	0.335
Triglycerides (mmol/L)	2.5 (1.1-15.8)	2.0 (1.2-3.1)	0.041
LDL-c (mmol/L)	3.9 ± 1.2	4.8 ± 1.3	0.025
HDL-c (mmol/L)	1.1 ± 0.4	1.3 ± 0.3	0.051
VLDL-c (mmol/L)	1.1 (0.5-7.6)	0.9 (0.6-1.3)	0.079
NEFA (mmol/L)	0.7 (0.3-4.3)	0.5 (0.2-1.1)	0.015
ApoB (g/L)	1.3 ± 0.3	1.4 ± 0.3	0.116
LDL size (nm)	25.4 ± 0.7	25.7 ± 0.4	0.184
CRP (mg/L)	1.6 (0.3-21.1)	1.5 (0.2-21.5)	0.901
25(OH)D (nmol/L)	48.2 ± 14.3	96.9 ± 11.7	0.000

P values according to T-test and Mann–Whitney test.

FCHL: Familial combined hyperlipidemia, BMI: body mass index, HbA1c: glicated haemoglobin, HOMA: homeostasis model assessment, LDL-c: low-density lipoprotein cholesterol, HDL-c: high-density lipoprotein cholesterol, VLDL-c: very low-density lipoprotein cholesterol, NEFA: non-esterified fatty acids, ApoB: apolipoprotein B, CRP: C-reactive protein, 25(OH)D: 25-hydroxyvitamin D.

Vitamin D deficiency = 25(OH)D ≤ 80 nmol/L.

### Longitudinal study

Twenty-three patients were included in the longitudinal study. Their mean age was 50.13 ± 10.2 years and 65.2% were men. Data are reported before and after 2.5 (1.9-12.9) months of treatment for their dyslipidemia. Three patients were treated with diet alone, 11 with statins, 5 with fibrates, 3 with statin plus fibrate and one patient with fibrate plus ezetimibe.

As expected, all lipid parameters improved after treatment. No significant changes were seen regarding anthropometric parameters and CRP levels (Table 3). However, 25(OH)D concentrations increased from 51.0 ± 31.3 to 58.9 ± 24.6 nmol/L (p = 0.022). The increase in 25(OH)D concentrations was higher in patients treated with a statin alone or in combination (7.5 (-10.6-46.5) vs. 1.45 (-17-7.1; p = 0.039). No differences were found between patients who received or not a fibrate. Changes in 25(OH)D concentrations did not correlate with changes in anthropometric measures, HOMA index, lipid parameters or CRP.

**Table 3:** Characteristics of patients with FCHL included in the longitudinal study before and after treatment for their dyslipidemia

	**Off- treatment**	**With treatment**	**P**
25(OH)D (nmol/L)	51.0 ± 31.3	58.9 ± 24.6	0.022
BMI (Kg/m^2^)	26.6 ± 2.6	26.6 ±2.6	0.778
Waist (cm)	92.7 ± 9.2	93.1 ± 8.8	0.518
HOMA	2.0 ± 1.1	2.2 ± 1.4	0.535
Total cholesterol (mmol/L)	7.4 (5.1-16.1)	5.1 (3.8-8.9)	0.000
Triglycerides (mmol/L)	2.8 (1.2-10.7)	1.9 (0.8-3.8)	0.000
LDL-c (mmol/L)	4.3 (1.4-7.8)	3.0 (2.0-5.8)	0.007
HDL-c (mmol/L)	1.2 ± 0.4	1.3 ± 0.3	0.000
VLDL-c (mmol/L)	1.3 (0.6-8.6)	0.9 (0.4-3.3)	0.000
NEFA (mmol/L)	0.6 (0.3-1.6)	0.6 (0.3-1.4)	0.313
ApoB (g/L)	1.4 ± 0.3	1.0 ± 0.2	0.000
LDLsize (nm)	25.3 (23.2-26.2)	25.7 (24.8-26.5)	0.058
CRP (mg/L)	2.0 (0.1-21.1)	1.3 (0.3-12.2)	0.823

P values according to paired T-test and Wilcoxon test.

FCHL: Familial combined hyperlipidemia, 25(OH)D: 25-hydroxyvitamin D, BMI: body mass index, HOMA: homeostasis model assessment, LDL-c: low-density lipoprotein cholesterol, HDL-c: high-density lipoprotein cholesterol, VLDL-c: very low-density lipoprotein cholesterol, LDL: low-density lipoprotein, NEFA: non-esterified fatty acids, ApoB: apolipoprotein B, CRP: C-reactive protein.

We analysed the time of the year when blood samples were obtained in order to detect possible influences of sun exposure on the changes observed in 25(OH)D concentrations. We considered two periods of sun exposure: a period of reduced sun exposure (November to April) and a period of maximum sun exposure (May to October) and we observed that 20 patients had both determinations, before and after treatment, during the same period of sun exposure. Only 3 patients had their first blood sample (off-treatment) taken during the period of less sun exposure and the second blood sample (after treatment) during the period of more sun exposure.

## Discussion

The main finding in the present study is that patients with FCHL had lower 25(OH)D concentrations that are related to lipid alterations and that treatment for dyslipidemia in our routine clinical practice was associated with an increase in 25(OH)D concentrations. These findings are novel and suggest that hypovitaminosis D is parallel to lipid alterations characteristic of FCHL.

In recent years, several reports have pointed out a possible association between vitamin D concentrations and dyslipidemia. In 1989, Wilczek et al. [28] found that heterozygotes with familial hypercholesterolemia suffered from vitamin D deficiency. Later on, associations of 25(OH)D with the different lipid parameters have been found in a wide range of populations. Melamed et al. [29] found an association between 25(OH)D and cholesterol concentrations in US participants of the National Health And Examination Survey (NHANES). Moreover, Lu et al. [30] and Lee et al. [14] found associations between 25(OH)D and HDL-c and triglyceride concentrations in Chinese and European populations, respectively. The widest study assessing the association between vitamin D and lipids included 108711 patients from a private clinical database in US; patients with optimal 25(OH)D concentrations had lower LDL-c and triglycerides and higher HDL-c concentrations than patients with suboptimal 25(OH)D concentrations [31]. In obese subjects, atherogenic dyslipidemia has been associated with hypovitaminosis D [32]. Finally, a meta-analysis of 22 cross-sectional studies concludes there is an association of vitamin D deficiency with an unfavourable lipid profile characterized by low HDL-c and high triglyceride concentrations [9]. Results from this meta-analysis, however, point out that not all studies adjusted their findings for possible confounders such as gender, age or BMI. Furthermore, they show discordant findings regarding the association of 25(OH)D concentrations with total cholesterol or LDL-c. Our study adds knowledge to the relation of vitamin D with lipid disorders by showing that FCHL, an inherited disease closely linked to the metabolic syndrome, is also associated with decreased 25(OH)D concentrations. Furthermore, and in accordance with previous studies, our patients with FCHL and vitamin D deficiency presented higher triglyceride and NEFA concentrations and lower LDL-c and HDL-c concentrations. Most of these associations persist after adjusting for BMI, suggesting a direct relationship between 25(OH)D and the lipid profile characteristic of atherogenic dyslipidemia.

Several authors have shown that statin use may be associated with beneficial effects on bone fracture [33]. Although the underlying mechanism for this association is unknown, the possibility that statins increase vitamin D concentrations cannot be ruled out. In fact, although results are heterogeneous, several studies have found a positive effect of statin use on vitamin D deficiency and some authors suggest that this effect might be through pleiotropic properties of statins and independently of their intrinsic effect on serum lipids [34-38]. Information regarding the effect of lipid-lowering drugs other than statins is scarce but ezetimibe and fibrates could also have an effect [39,40]. In our study, treatment for dyslipidemia was accompanied by an increase in 25(OH)D concentrations of 7.86 nmol/L. As our patients were treated in a clinical practice setting, the type and dose of lipid lowering medications was heterogeneous; most patients received statins, but a considerable number were also treated fibrates in monotherapy or combined with statins. BMI did not change during the longitudinal study and it is interesting to note that changes in lipid parameters did not correlate with changes in 25(OH)D concentrations, suggesting that the effect of lipid-lowering therapy on vitamin D metabolism may be through a lipid independent pathway. In fact, although our results should be treated with caution due to the small number of subjects included, we have found that patients prescribed statins had greater increases in vitamin D concentrations than patients not taking statins. Thus, a pleitropic effect of lipid-lowering drugs, specially statins, on vitamin D concentrations cannot be ruled out.

The main strength of our work is the adjustment for potential confounders such as BMI and seasonality, when considering differences in vitamin D concentrations in cross-sectional analysis. Furthermore, patient selection excluded subjects taking vitamin D derivatives, and, as all patients were Caucasian, a possible effect of different ethnicities on vitamin D metabolism was eliminated. In the longitudinal study, as most determinations were performed at the same sunlight period, the possible effect of changes in seasonality was minimized. The main limitation of our work is patient number and the fact that the cross-sectional design of the analysis does not enable to draw causative relations between FCHL and vitamin D. However, the fact that treatment for dyslipidemia is associated with an increase in 25(OH)D concentrations, independently of lipid changes suggests that the association of FCHL with vitamin D is complex and not only driven by lipid parameters. Reference group selection included patients with overweight or with increased CRP; however, this should not be taken as a limitation as, if more strict selection criteria were applied, probably differences found would have been magnified. Finally, we cannot exclude that changes in lifestyle habits that accompany treatment initiation for dyslipidaemia, could partly account for vitamin D changes, so further studies are needed to confirm our findings.

## Conclusions

The main conclusion of our work is that FCHL is associated with decreased vitamin D concentrations that are related to features characteristic of atherogenic dyslipidemia. Moreover, treatment for dyslipidemia improves vitamin D status through an unknown mechanism. As vitamin D deficiency seems to play an important role in cardiovascular health, the decreased vitamin D concentrations found in patients with FCHL may contribute to the development of atherosclerosis in patients with this primary hyperlipidemia. Future studies are needed to replicate these data in larger populations and to elucidate the mechanisms that are involved in this association.

## Abbreviations

ANCOVA: Analysis of covariance; ApoB: Apolipoprotein B; BMI: Body mass index; CRP: C-reactive protein; CV: Coefficient of variation; FCHL: Familial combined hyperlipidemia; HDL-c: High-density lipoprotein cholesterol; HOMA: Homeostasis model assessment; LDL: Low-density lipoprotein; LDL-c: Low-density lipoprotein cholesterol; NEFA: Non-esterified fatty acids; NHANES: National Health And Examination Survey; S.E.M: Standard error of the mean; VLDL-c: Very-low density lipoprotein cholesterol; 25(OH)D: 25-hydroxyvitamin D.

## Competing interests

The authors declared no conflicts of interest related to this manuscript.

## Authors’ contributions

IM and JS-H participated in the design of the study and carried out the statistical analysis and drafting of the manuscript. JLS-Q and JR carried out analysis of data and drafting of the manuscript. AL and AP participated in the design of the study and have given critical revision of the manuscript and final approval. All authors read and approved the final manuscript.
